# No consensus on gestational diabetes mellitus screening regimes in Sweden: pregnancy outcomes in relation to different screening regimes 2011 to 2012, a cross-sectional study

**DOI:** 10.1186/1471-2393-14-185

**Published:** 2014-05-31

**Authors:** Maria Lindqvist, Margareta Persson, Marie Lindkvist, Ingrid Mogren

**Affiliations:** 1 Department of Clinical Sciences, Obstetrics and Gynecology, Umeå University, Umeå, Sweden; 2 Dalarna University, School of Health and Social studies, Falun, Sweden; 3 Department of Statistics and Department of Public Health and Clinical Medicine, Umeå University, Umeå, Sweden

## Abstract

**Background:**

Although associated adverse pregnancy outcomes, no international or Swedish consensus exists that identifies a cut-off value or what screening method to use for definition of gestational diabetes mellitus. This study investigates the following: *i)* guidelines for screening of GDM; *ii)* background and risk factors for GDM and selection to OGTT; and *iii)* pregnancy outcomes in relation to GDM, screening regimes and levels of OGTT 2 hour glucose values.

**Methods:**

This cross-sectional and population-based study uses data from the Swedish Maternal Health Care Register (MHCR) (2011 and 2012) combined with guidelines for GDM screening (2011–2012) from each Maternal Health Care Area (MHCA) in Sweden. The sample consisted of 184,183 women: 88,140 in 2011 and 96,043 in 2012. Chi-square and two independent samples t-tests were used. Univariate and multivariate logistic regression analyses were performed.

**Results:**

Four screening regimes of oral glucose tolerance test (OGTT) (75 g of glucose) were used: A) universal screening with a 2-hour cut-off value of 10.0 mmol/L; B) selective screening with a 2-hour cut-off value of 8.9 mmol/L; C) selective screening with a 2-hour cut-off value of 10.0 mmol/L; and D) selective screening with a 2-hour cut-off value of 12.2 mmol/L. The highest prevalence of GDM (2.9%) was found with a 2-hour cut-off value of 8.9 mmol/L when selective screening was applied. Unemployment and low educational level were associated with an increased risk of GDM. The OR was 4.14 (CI 95%: 3.81-4.50) for GDM in obese women compared to women with BMI <30 kg/m^2^. Women with non-Nordic origin presented a more than doubled risk for GDM compared to women with Nordic origin (OR = 2.24; CI 95%: 2.06-2.43). Increasing OGTT values were associated with increasing risks of adverse pregnancy outcomes.

**Conclusions:**

There was no consensus regarding screening regimes for GDM from 2011 through 2012 when four different regimes were applied in Sweden. Increasing levels of OGTT 2-hour glucose values were strongly associated with adverse pregnancy outcomes. Based on these findings, we suggest that Sweden adopts the recent recommendations of the International Association of Diabetes and Pregnancy Study Group (IADPSG) concerning the performance of OGTT and the diagnostic criteria for GDM.

## Background

Gestational diabetes mellitus (GDM), one of the most common metabolic disorders complicating pregnancy, is defined as any degree of glucose intolerance with onset or first recognition during pregnancy, and that is not considered manifest diabetes mellitus type 2 [1]. Risk factors for GDM are family history of diabetes mellitus type 2 (DM2), previous GDM, macrosomic infant (defined as 4500 grams or more), BMI ≥30 kg/m^2^[2], unexplained intrauterine fetal death, maternal age ≥35 years, and immigrant status [3-5]. The results from the Hyperglycemia and Adverse Pregnancy Outcome (HAPO) study show that higher maternal glucose levels are associated with increased macrosomia, caesarean sections and neonatal hypoglycemia [6]. Compared to normoglycemic pregnancies, women with GDM are associated with a seven-fold increased risk of DM2 later in life [7]. Offspring of women with GDM pregnancies have increased risk of obesity, glucose intolerance, and diabetes mellitus in puberty or early adulthood, all conditions included in metabolic syndrome [8,9].

The global prevalence of GDM is 7% (1-14% depending on diagnostic tests and the population studied) [10]. Applying the diagnostic criteria of the International Association of Diabetes and Pregnancy Study Group (IADPSG) and the international multi-centre HAPO has demonstrated significant variability of prevalences of GDM among participating countries and even among participating study centres within the same country [11]. From a global perspective, Sweden is considered a low risk country for GDM with an annual prevalence of 1 to 2.6% of this pregnancy-related disorder [12,13]. There is, however, no international consensus regarding how women should be screened for GDM, whether screening should be undertaken universally, or whether women who present risk factors [14] should undergo screening, i.e. selective screening. This lack of consensus persists even though it is recognized that adverse pregnancy outcomes are associated with GDM and a diagnosis of GDM results in increased medical surveillance for mother and fetus during pregnancy [15]. Therefore, it is impossible to estimate the true prevalence of GDM.

In Sweden, there is no national consensus with respect to GDM screening [16]. The International Association of Diabetes and Pregnancy Study Group (IADPSG) has presented recommendations for thresholds of GDM based on the epidemiological HAPO study [17], and these recommendations have also been adopted by the World Health Organization (WHO) [18]. The proposed criteria for GDM when using a 75-gram oral glucose tolerance test (OGTT) include a fasting plasma glucose of 5.1-6.9 mmol/L (92–125 mg/dL) or a 1-hour plasma glucose of 10.0 mmol/L (180 mg/dL) or a 2-hour plasma glucose of 8.5 mmol/L (153 mg/dL) [17].

Antenatal care (ANC) in Sweden is free and currently organized in 43 different areas called Maternal Health Care Areas (MHCA) characterized by geographic boundaries corresponding mainly to the Swedish counties. A consultant obstetrician and a consultant midwife are responsible for the guidelines regarding maternal and fetal surveillance and medical management within each MHCA. National guidelines of surveillance during pregnancy in antenatal care have been suggested by the professional associations of obstetricians and midwives [19]; however, local guidelines may differ between MHCA [16,19]. The overall aim of antenatal care in Sweden is to achieve “good sexual and reproductive health for the whole population”, a goal that is similar to the WHO’s guidelines for sexual and reproductive health. Almost all pregnant women in Sweden register in Swedish antenatal care [19].

In the Nordic countries, there is a unique opportunity to use population-based national registers for research. Due to these unique personal data registers, data may be linked, and individuals and families can be identified in several generations. The national registers are creating unique possibilities and are a valuable source of information and matrix for clinical trials [20]. The Swedish Maternal Health Care Register (MHCR) is a national health register, and data have been collected since 1999 by midwives in antenatal care. The MHCR underwent a major revision of the included variables between 2007 and 2009, and a new version of the register was launched in 2010. The MHCR is currently monitoring around 85% of all pregnant women (personal correspondence).

There is an ongoing discussion internationally regarding the diagnostics of GDM. However, suggestions of lowering cut-off values for GDM diagnosis, the situation of GDM screening in Sweden has not changed in the last decade and there are several regimes of screening available in clinical practice. Hence, there is an opportunity to study the outcomes of different screening regimes as to add further information to the knowledge of outcomes in relation to screening regimes.

This study, which includes data on deliveries in Sweden from January 1^st^ 2011 to December 31^st^ 2012, investigated the following: *i)* guidelines for screening of GDM; *ii)* background and risk factors for GDM and selection to OGTT; and *iii)* pregnancy outcomes in relation to GDM, screening regimes and levels of OGTT 2 hour glucose values.

## Methods

This cross-sectional and population-based study uses data retrieved from the Swedish Maternal Health Care Register for 2011 and 2012 in combination with local guidelines for screening of GDM collected from each MHCA in Sweden.

All women, irrespective of single birth or multiple births, with data registered in the MHCR between 2011 and 2012 were included in the study. The coverage of registered deliveries in the MHCR was estimated to be 81% for 2011 and 85% for 2012 (personal correspondence). The following variables retrieved from the MHCR were included in the study: country of origin, maternal age, parity, maternal height, maternal weight, body mass index, smoking, level of education, self-reported health, number of visits to antenatal care, use of professional interpreter at visits in ANC, treatment for psychological ill-health during pregnancy, GDM, OGTT, gestational age, delivery mode, birth weight, small for gestational age (SGA), appropriate for gestational age (AGA), and large for gestational age (LGA) [21].

Local guidelines for screening of GDM were collected from all MHCA in Sweden (n = 43) through an initial e-mail request between September and October 2012. The e-mail requesting local guidelines was sent to the consultant midwife in each MHCA. The majority of guidelines were collected during the first round. For those MHCA that did not respond to the first e-mail, the consultant midwife in each MHCA was contacted by telephone or by another e-mail. Finally, guidelines for screening for GDM were collected from all MHCA in Sweden. Similarities and differences in the guidelines were compared. All local guidelines for screening GDM were unchanged during the study period (2011 to 2012).

### Selective screening for GDM based on risk factors

Since 1990, Sweden has officially used the recommendations developed by the European Association for the Study of Diabetes for screening for GDM. Both universal and selective screening regimes stipulate a 75-g glucose load and the two-hour value of the capillary plasma glucose for the diagnosis. However, there is no national consensus regarding the threshold for definition of GDM in Sweden, so it varies among the different MHCA. Presently, there are two national approaches for screening for GDM: one universal screening approach where all pregnant women are offered an OGTT and one selective screening approach based on specified risk factors in the local guideline. The risk factor approach (i.e., selective screening) is used by approximately 89% of the MHCA in Sweden (personal communication). The risk factors that indicate a need for screening for GDM include family history of diabetes mellitus type 2 (DM2), GDM, macrosomic infant (defined as 4500 grams or more) or stillbirth in previous pregnancy, BMI ≥30 kg/m^2^, and non-European nationality. The risk factors during pregnancy indicating an OGTT are accelerating fetal growth, polyhydramniosis, and elevated random capillary plasma glucose. Both universal and selective screening regimes for the diagnostic procedures stipulate the use of a 75-g glucose load and the 2-hour value of the capillary plasma glucose. One-third of the MHCA also uses fasting glucose as a diagnostic criterion for GDM.

### Definitions of background and outcome variables

*Maternal age* was defined as age at delivery. *Parity* was defined as total number of deliveries (including the index pregnancy in the register). *Maternal height* (cm), and *maternal weight* in early pregnancy (kg) were self-reported by the pregnant woman. *Body mass index (BMI)* was calculated with the formula BMI kg/m^2^. The different BMI groups were defined according to WHO’s definition of BMI: underweight: <18.5 kg/m^2^; normal range: 18.5-24.99 kg/m^2^; overweight: 25–29.99 kg/m^2^; obesity class 1: 30–34.99 kg/m^2^; obesity class 2: 35–39.99 kg/m^2^; and obesity class 3: ≥40 kg/m^2^. *Smoking* at three months before pregnancy and at the first antenatal visit was reported. *Level of education* was defined as elementary school, high school, and university. *Self-reported health* was reported by the woman during early pregnancy and divided into five categories: very good, good, either good or poor, poor, and very poor health*. Gestational diabetes mellitus (GDM)* was defined as any degree of glucose intolerance with onset or first recognition during pregnancy*.* The cut-off values – 8.9 mmol/L, 10.0 mmol/L, and 12.2 mmol/L – for the diagnosis of GDM were determined by the MHCA. *OGTT* (75 g 2-two-hour oral glucose tolerance test) was used to diagnose GDM in all parts of Sweden. For analysis, the OGTT glucose values were categorised into two different sets. The first set of categories was as follows: <7.5 mmol/L, 7.5-8.8 mmol/L, 8.1-8.9 mmol/L, 9.0-9.9 mmol/L, 10.0-12.1 mmol/L, and ≥12.2 mmol/L. The second set used the categories defined by IADPSG, which define a normal OGTT as a glucose value of <8.5 mmol/L, GDM (i.e., glucose value) between 8.5 and 11.0 mmol/L, and manifest diabetes mellitus type 2 (i.e., glucose value ≥11.1 mmol/L) [17]. *Delivery mode* was reported as either as vaginal non-instrumental, vaginal instrumental, elective caesarean section, or emergency caesarean section. *Birth weight* was reported in grams. In calculations of birth weight, the birth weight of single births were included as well as birth weight of first child of duplex or triplex pregnancy. *Small for gestational age (SGA)*, *appropriate for gestational age (AGA)*, and *large for gestational age (LGA)* were calculated using Marsal’s curve [21].

Ethical approval from the Ethical Review Board in Umeå was granted 2012 (Dno. 2012-407-3IM).

### Statistical analysis

Two-independent samples t-tests were used to test differences of parametric data, and non-parametric two-independent samples tests were used to test differences of categorical variables. Univariate and multivariate logistic regression analyses were performed and presented with odds ratios (OR) and their 95% confidence intervals (CI) where different models were demonstrated. The population attributable proportion (PAP) was calculated for specified exposures using the formula PAP = p(RR-1)/[1 + p(RR-1)], where PAP is the proportion of cases in the population that should not have occurred had the exposed had the incidence of the unexposed. Statistical analysis was done using SPSS version 19.

## Results

The total sample consisted of 184,183 deliveries (88,140 in 2011 and 96,043 in 2012). Mean maternal age and mean parity at delivery was 30.25 years and 1.8, respectively (Table 1). Table 1 shows the background characteristics for the whole sample as well as for each year (2011 and 2012). Test of difference was calculated for 2011 vs. 2012 for all variables. The following significant p-values were found comparing the variables for the year 2011 and the year 2012: maternal age in years (p = 0.032), weight (0.005), and BMI (p = 0.001). Regarding performed OGTT, 2011 vs. 2012 significant p-values were found for the variables weight (p = 0.001), BMI (0.001), educational level (p = 0.003), employment status (p = 0.012), number of visits to ANC (p = 0.011), SRH (p = 0.001), reported smoking three months before pregnancy (p = 0.001), and reported smoking at first visit (p = 0.001). The variables are presented in Table 1.

**Table 1:** Characteristics of subjects and test of difference between specified categories

**Variables**^**a**^	**All subjects 2011-2012 N (%)**	**Subjects 2011 n (%)**	**Subjects 2012 n (%)**	**OGTT 2011-2012 N (%)**	**GDM 2011-2012 N (%)**
**Maternal (yrs)**	184130 (99.8)	88016 (99.7)	96025 (99.9)	38302 (99.8)	2579 (99.8)
Mean(SD)	30.25 (5.3)	30.27 (5.3)	30.23 (5.3)	30.36 (5.4)	31.86 (5.5)
Min-Max	13-57	13-53	13-57	15-54	16-53
**Maternal age**					
≤19	2688 (1.5)	1369 (1.6)	1319 (1.4)	469 (17.6)	23 (0.9)
20-24	25212 (13.7)	11990 (13.8)	13222 (13.8)	5293 (21.2)	239 (1.0)
25-29	53971 (29.3)	25570 (29.0)	28393 (29.6)	11257 (21.0)	618 (1.1)
30-34	61706 (33.5)	29463 (33.4)	32243 (33.6)	12432 (20.3)	837 (1.4)
35-39	33225 (18.0)	16215 (18.4)	17010 (17.7)	7130 (21.5)	642 (1.9)
≥40	738 (4.0)	3490 (4.0)	3838 (4.0)	1748 (24.0)	220 (3.0)
**Parity**	181292 (98.4)	86189 (96.0)	95103 (98.2)	37603(96.8)	2548 (97.3)
1	79250 (43.7)	37569 (43.6)	41681 (43.8)	15493 (19.7)	949 (1.2
2	67902 (37.5)	32340 (37.5)	35562 (37.4)	14130 (20.9)	861 (1.3)
≥3	34140 (18.8)	16280 (18.9)	17860 (18.8)	7980 (22.8)	738 (2.9)
**Gest age**^**bc**^	180822 (98.2)	86434 (97.9)	94388 (98.1)	37684 (97.2)	2513 (97.6)
**Pre-term**	9838 (5.4)	4689 (5.4)	5149 (5.5)	1914 (19.6)	209 (2.1)
**Term**	156599 (86.6)	74874 (86.6)	81725 (86.6)	32714 (21.0)	2208 (1.4)
**Post-term**	14385 (8.0)	6871 (8.0)	7514 (8.0)	3056 (21.4)	96 (0.7)
**Weight (kg)**	179343 (97.4)	85359 (97.9)	93984 (97.9)	37062 (96.8)	2510 (97.3)
Mean (SD)	68.50 (13.7)	68.40 (13.6)	68.59 (13.7)	75.45 (17.7)	7704 (18.6)
Min-Max	29-183	29-183	30-183	36-183	40-163
**Height** (cm)	180124 (97.8)	85764 (97.3)	94360 (98.2)	37762 (97.3)	2519 (97.7)
Mean (SD)	166.2 (6.5)	166.2 (6.5)	166.2 (6.5)	166.2 (6.6)	163.8 (6.8)
Min-Max	113-196	113-193	113-196	133-195	133-187
**BMI (kg/m**^**2**^**)**^**d**^	178723 (97.0)	85046 (97.5)		36967 (96.8)	2496(96.8)
Mean (SD)	24.78 (4.6)	24.74 (4.6)	24.82 (4.7)	27.29 (6.1)	28.70 (6.3)
Min-max	13.6-62.6	13.8-62.1	13.6-60.0	13.6-62.1)	16.0-60.0
**BMI early pregnancy**	178723 (97.0)	85046 (97.5)	93677 (96.7)	36967 (96.5)	2496(96.8)
<18.5	4329 (2.4)	2031 (2.4)	2298 (2.5)	586 (13.6)	23 (0.5)
18.5-24.99	1056679(59.1)	50622(59.5)	55057 (58.8)	15633 (14.9)	789 (0.7)
25-29.99	45667 (25.6)	21641 (25.4)	24026 (25.6)	9285 (20.4)	754 (1.7)
30-34.99	16126 (9.0)	7537 (8.8)	8589 (9.2)	6793 (42.4)	511 (3.2)
35-39.99	5117 (2.9)	2379 (2.8)	2738 (2.9)	3327 (65.7)	285 (5.8)
≥40	1805 (1.0)	836 (1.0)	969 (1.0)	1313 (73.3)	134 (7.4)
**Educational level**	151600 (82.3)	70638 (79.5)	80962 (84.0)	32183 (82.3)	2003 (76.5)
Elementary school	13532 (8.9)	6662 (9.4)	6870 (8.5)	3239 (24.1)	309 (2.4)
High school	60481 (39.9)	27821 (39.4)	32660 (40.3)	14263 (23.7)	904 (1.5)
University	77587 (51.2)	36155 (51.2)	41432 (51.2)	14681 (19.0)	790 (1.0)
**Employment status**	179628 (97.5)	85165 (94.6)	94463 (98.0)	37330 (96.1)	2501 (95.6)
Employed^e^	159566 (88.8)	75669 (88.8)	83897 (88.8)	32805 (21.9)	2057 (1.6)
Unemployed^f^	20062 (11.2)	9496 (11.2)	10566 (11.2)	4525 (22.9)	444 (2.4)
**Country of origin**	184132 (100)	88140 (100)	96043 (100)	38308 (97.6)	2579 (97.2)
Sweden	144563 (78.5)	70376 (79.8)	74187 (77.2)	29637 (20.6)	1613 (1.1)
Nordic countries^g^	1445 (0.8)	613 (0.7)	832 (0.9)	350 (24.6)	23 (1.7)
Other countries	34134 (18.5)	17151 (19.5)	21024 (21.9)	8318 (22.4)	943 (2.8)
**Visits at ANC**^**h**^	182137 (98.9)	86822 (98.6)	95315 (99.2)	37917(99.0)	2534 (98.3)
Mean:	8.8 (2.4)	8.8 (2.4)	8.7 (2.7)	9.0 (2.4)	9.3 (3.0)
Max-min:	1-29	2-26	1-24	1-29	1-26
**SRH**^**i**^	153227 (83.2)	70633 (79.1)	82594 (85.7)	32442 (83.3)	2102 (80.3)
Very good	44310 (28.9)	19838 (28.1)	24472 (29.6)	8484 (19.2)	422 (1.0)
Good	89740 (58.6)	41299 (58.5)	48441 (58.6)	19650 (22.0)	1317 (1.5)
Either good or poor	13755 (9.0)	6793 (9.6)	6962 (8.4)	3142 (23.0)	260 (1.9)
Poor	4371 (2.9)	2174 (3.1)	2197 (2.7)	950 (21.8)	82 (1.9)
Very poor	1051 (0.7)	529 (0.7)	522 (0.6)	216 (20.7)	21 (2.0)
**Smoking 3 months**^**j**^	182532 (99.1)	75316 (98.8)	81797 (99.2)	38305 (97.5)	2579(97.6)
No smoking	157113 (85.2)	75316 (85.5)	81797 (86.1)	31718 (20.3)	2167(1.4)
Smoking	25419 (14.8)	12134 (14.5)	13285 (13.9)	5976 (23.7)	387 (1.6)
**Smoking first visit**	182619 (99.0)	84134 (94.2)	96043 (98.5)	38305 (97.6)	2579 (97.6)
No smoking	172082 (94.3)	82451 (94.2)	89631 (94.1)	35139 (20.5)	2380 (1.5)
Smoking	10537 (5.7)	5062 (5.7)	5475 (5.9)	2578 (24.6)	174 (1.7)

^a^For each specified variable, n and% are presented.

^b^Gestational age calculated using the WHO’s guidelines.

^c^Pre-term = 22 + 0-36 + 6, Term = 37 + 0-41 + 6, Post-term = 42 + 0–43 + 6 (weeks).

^d^Body mass index (BMI).

^e^Employed includes employed, student, and parental leave.

^f^Unemployed includes unemployed, sick leave, and other.

^g^Norway, Denmark, Finland, and Iceland.

^h^Antenatal care unit (ANC).

^i^Self-rated health (SRH).

^j^Smoking three months before pregnancy.

### Four screening regimes for diagnostics of GDM in Sweden

Nationally, four GDM screening regimes were used between 2011 and 2012, using different capillary plasma glucose values for the diagnosis of GDM. Accordingly, there was no national consensus regarding screening and diagnosis of GDM in Sweden (Table 2). Four schemes for GDM screening were followed: A) universal screening with a 2-hour cut-off value of 10.0 mmol/L; B) selective screening with a 2-hour cut-off value of 8.9 mmol/L; C) selective screening with a 2-hour cut-off value of 10.0 mmol/L; and D) selective screening with a 2-hour cut-off value of 12.2 mmol/L. The OGTT 2-hour cut-off value of 12.2 mmol/L as diagnosis for GDM in the fourth category was regarded as diagnosis of manifest diabetes mellitus in the majority of the MHCA in Sweden. Most pregnant women (56.8%) in Sweden underwent selective screening of GDM with a 2-hour cut-off glucose value of 10.0 mmol/L. Fewer pregnant women (11.3%) were offered universal screening with the same criteria for diagnosis (10 mmol/L). In total, 88.7% of pregnant women in Sweden underwent selective screening. The background factors family history of diabetes mellitus type 2, previous GDM, macrosomic infant (≥4.5 kg), and BMI >30 kg/m^2^ were used by 81% of the MHCA as indicators for OGTT during pregnancy. Other indicators were unexplained intrauterine fetal death, maternal age more than 35 years, and immigrant status. Among the MHCA, only four had immigrant status as an indicator for OGTT. A vast majority of MHCA (81%) used BMI >30 or 35 as an indicator for OGTT. The most prevalent risk indicator for OGTT was elevated random plasma glucose (8.0-9.0 mmol/L), which was presented in all the MHCA using selective screening.

**Table 2:** Maternal characteristics and specific outcomes in relation to four screening regimes for gestational diabetes mellitus (GDM) in Sweden between 2011 and 2012

**Variables**	**All**	**MA**^**a **^**(yrs)**	**Height (cm)**	**BMI**^**b**^	**BMI**^**b**^	**OGTT**^**c**^	**GDM**
	**n (%)**	**Mean**	**Mean**	**Mean**	**≥30**	**n (%)**	**n (%)**
		**Min-max**	**Min-max**	**Min-max**	**n (%)**		
**A. Universal screening** 10.0 mmol/L^d^	20822 (11.3)	30.0	166.4	25.04	2784 (14.1)	19294 (93.6)	456 (2.2)
		15–49	140–190	13.63–55.98			
**B. Selective screening** 8.9 mmol/L^d^	8634 (4.7)	29.8	166.4	24.99	1110 (13.4)	1674 (19.6)	252 (2.9)
		15–49	135–190	13.82–56.65			
**C. Selective screening** 10.0 mmol/L^d^	104688 (56.8)	29.8	166.2	25.04	14523 (14.2)	13934 (13.4)	1494 (1.4)
		13–54	113–195	13.90–62.06			
**D. Selective screening** 12.2 mmol/L^d^	50039 (27.2)	31.4	166.2	24.11	4631 (9.6)	3403 (6.8)	377 (0.8)
		14–57	123–196	13.97–56.40)			

^a^Maternal age (MA).

^b^Body mass index (BMI) kg/m^2^.

^c^Oral glucose tolerance test (OGTT).

^d^Cut-off value.

### Prevalences of OGTT and GDM

Table 2 presents maternal background characteristics, prevalences of OGTT, GDM, and birth weight (mean, minimum, and maximum) in relation to current regimes of screening of GDM between 2011 and 2012. As expected, the prevalence of OGTT was highest (93.6%) in the MHCA that offered pregnant women universal screening of GDM, and the prevalence of OGTT was lowest (6.8%) in the screening category with a 2-hour cut-off value of 12.2 mmol/L as criteria for GDM (Table 2). The lowest prevalence of OGTT was for women younger than 19 years old (17.6%). The highest prevalence was for women 40 years old or older (24.0%) (Table 1). As body mass index increased, the prevalence of OGTT increased. The lowest prevalence was for women in the lowest BMI category (BMI < 18.5 kg/m^2^; 13.6%), and the highest prevalence was for women with a BMI between 35 and 39.99 kg/m^2^ (65.7%) and women with BMI 40 or higher (73.3%) (Table 1). The highest prevalence of GDM (2.9%) was found in the areas where selective screening was applied with an OGTT 2-hour value of 8.9 mmol/L as criteria for diagnosis of GDM (Table 2). As shown in Table 1, the prevalence of GDM increased with increasing values in categories of maternal age, BMI, and parity. Furthermore, the prevalence of GDM increased in women who had a low educational level, who were unemployed, and who rated their health as “either good or poor” or “very poor” compared to women with higher educational level, women who were employed, and women who rated their health as “good or very good” (Table 1).

### Population attributable proportion

To calculate the population attributable proportion, we selected the two screening regimes universal screening “A” (Table 2) and selective screening “C” (Table 2) (i.e., two categories using the same 2-hour capillary plasma glucose value of 10 mmol/L for diagnosis of GDM). For each screening regime, the PAP for GDM was calculated for the exposures obesity, increased maternal age (i.e., maternal age 35 years or more), and non-Nordic origin. For the screening regime “A”, 20%, 14%, and 22% of GDM cases could be attributed to obesity, increased maternal age, and non-Nordic origin, respectively. The corresponding PAPs for screening regime “C” were 31%, 17%, and 19% of GDM cases that could be attributed to obesity, increased maternal age, and non-Nordic origin, respectively.

### Maternal characteristics and pregnancy outcomes in relation to screening regimes

Maternal characteristics and pregnancy outcomes such as maternal age, maternal BMI, birth weight, large for gestational age, and small for gestational age are presented in Table 1 for the four different screening regimes and in relation to the subcategories of GDM and non-GDM within each screening regime. The levels of maternal BMI among non-GDM cases were similar between regimes with the exception of non-GDM cases in the category where selective screening with a cut-off value of 12.2 mmol/L was applied, which presented a lower BMI-value (Table 3). Overall, the highest prevalence of LGA cases (20.6%) was found among women with GDM in the MHCA where selective screening and a 2-hour cut-off value of 12.2 mmol/L for diagnosis of GDM was applied (Table 3).

**Table 3:** Maternal characteristics and specified pregnancy outcomes in relation to categories of universal and selective screening regimes for gestational diabetes mellitus (GDM) between 2011 and 2012

**Variables**	**Maternal age (years)**	**Maternal BMI**^**a**^	**Birth weight (grams)**	**LGA**^**b**^	**LGA/non LGA**	**SGA**^**c**^	**SGA/non LGA**
	**Mean**	**Mean**	**Mean**	**n (%)**	**p-value**		**p-value**
**Universal screening 10.0 mmol/L**^**d**^				890 (4.4)		478 (2.4)	
GDM	31.58	27.63	3540	46 (10.5)	<0.001	17 (3.9)	0.121
Non GDM	29.98	24.98	3533	844 (4.3)		59 (2.3)	
**Selective screening 8.9 mmol/L**^**d**^				382 (4.7)		218 (2.7)	
GDM	31.67	28.27	3567	25 (10.4)	<0.001	7 (2.9)	0.588
Non GDM	29.72	24.89	3555	352 (4.4)		210 (2.6)	
**Selective screening 10.0 mmol/L**^**d**^				4375 (4.3)		2586 (2.5)	
GDM	31.71	29.22	3636	215 (14.9)	<0.001	19 (1.3)	0.063
Non GDM	29.77	24.98	3534	4184 (4.1)		2561 (2.5)	
**Selective screening 12.2 mmol/L**^**d**^				1903 (4.0)		1289 (2.7)	
GDM	32.94	27.98	3610	84 (20.6)	<0.001	14 (3.8)	0.058
Non GDM	31.39	24.08	3506	1819 (3.9)		1273 (2.7)	

^a^Body mass index (BMI) kg/m^2^.

^b^Large for gestational age (LGA).

^c^Small for gestational age (SGA).

^d^OGTT 2-hour cut-off value.

### Pregnancy outcomes in relation to OGTT 2-hour glucose values

Increasing OGTT 2-hour glucose values were associated with increasing prevalences of adverse pregnancy outcomes such as instrumental vaginal delivery, caesarean section (elective CS as well as emergency CS), and large for gestational age (Table 4). Prevalences of LGA ranged from 5.7% (OGTT 2-hour glucose value of ≤7.5 mmol/L) to 15.6% (OGTT 2-hour glucose value ≥12.2 mmol/L). Prevalences of SGA were fairly similar in the different categories of OGTT values, ranging from 2.0 to 2.7% (Table 4). Furthermore, we categorized the OGTT 2-hour glucose values according to the criteria defined by IADPSG and WHO. The same pattern as in Table 4 was seen with increasing prevalences of similar adverse pregnancy outcomes (Table 5).

**Table 4:** Oral glucose tolerance test (OGTT) 2-hour values in categories in relation to specified pregnancy outcomes between 2011 and 2012

**OGTT values in categories**	**All**	**Birth weight (grams)**	**LGA**^**a**^	**SGA**^**b**^	**Vaginal non instrumental**	**Vaginal instrumental**	**CS**^**d**^	**Elective CS**	**Emergency CS**
**(mmol/L)**	**n (%)**	**Mean min-max**	**n (%)**	**n (%)**	**Delivery n(%)**	**Delivery n (%)**	**n (%)**	**n (%)**	**n (%)**
<7.5	26307 (71.6)	3594	1445 (5.7)	590 (2.4)	20082 (76.5)	1781 (6.8)	4388 (16.7)	1928 (7.3)	2455 (9.3)
		300–5890							
7.5–8.0	3644 (9.9)	3663	316 (9.0)	68 (2.1)	2646 (72.7)	264 (7.2)	732 (20.1)	302 (8.3)	430 (11.8)
		345–5940							
8.1–8.9	3576 (9.7)	3682	355 (10.3)	64 (2.0)	2537 (71.1)	262 (7.3)	770 (21.6)	315 (8.8)	455 (12.7)
		990–6020							
9.01–9.9	1504 (4.1)	3640	177 (12.2)	31 (2.4)	1024 (68.4)	105 (7.0))	369 (24.1)	157 (10.4)	211 (14.1)
		491–6270							
10.0–12.1	1411 (3.8)	3620	198 (14.8)	30 (2.5)	917 (65.2)	101 (7.2)	388 (27.6	174 (12.4)	214 (15.4)
		512–5955							
≥12.20	310 (0.8)	3619	46 (15.6)	7 (2.7)	188 (60.8)	24 (7.8)	97 (31.4)	44 (12.7)	57 (17.8)
		1103–5540							

^a^Large for gestational age (LGA).

^b^Small for gestational age (SGA).

^c^Vacuum extraction or forceps.

^d^Ceasarean section (CS).

**Table 5:** Oral glucose tolerance test (OGTT) 2-hour values in categories in relation to specified pregnancy outcomes between 2011 and 2012

**OGTT values in categories**	**All**	**Birth weight (grams)**	**LGA**^**a**^	**SGA**^**b**^	**Vaginal non instrumental**	**Vaginal instrumental**	**CS**^**d**^	**Elective CS**	**Emergency CS**
**(mmol/L)**	**n (%)**	**Mean min-max**	**n (%)**	**n (%)**	**Delivery n (%)**	**Delivery n (%)**	**n (%)**	**n (%)**	**n (%)**
<8.5	31974 (87.0)	3605	1940 (6.2)	696 (2.2)	24162 (75.7)	2201 (6.9)	5548 (17.4)	2392 (7.8)	3156 (9.6)
		300–5964							
8.5–11.0	4082 (11.1)	3661	474 (11.8)	76 (1.9)	2799 (68.8)	285 (11.2)	987 (24.2)	433 (10.6)	554 (13.6)
		491–6270							
≥11.1	696 (1.9)	3646	124 (18.3)	18 (2.7)	433 (62.5)	51 (7.4)	209 (30.2)	95 (11.9)	114 (18.3)
		1103–5955							

^a^Large for gestational age (LGA).

^b^Small for gestational age (SGA).

^c^Vacuum extraction or forceps.

^d^Ceasarean section (CS).

### Gestational diabetes mellitus in relation to maternal characteristics

Table 6 presents univariate and multivariate logistic regression analyses for the outcome GDM in relation to non-GDM for different background variables such as BMI, maternal age, country of origin, employment status, and educational level. Among the background variables investigated, obesity (defined as BMI >30 kg/m^2^) demonstrated the strongest impact on risk of GDM (Table 6). The OR for GDM was 4.14 (95% CI 3.81-4.50) for women with obesity compared to women with BMI less than 30. When adjusting for age, country of birth, employment status, and educational level, the OR was moderately changed with an OR of 3.66 (95% CI 3.31-4.01). Women with a non-Nordic origin had more than twice the risk for GDM in relation to women with Nordic origin (Table 6).

**Table 6:** **Univariate and multivariate logistic regression analysis**^**a b **^**for gestational diabetes mellitus (GDM) in relation to non-GDM for specified variables between 2011 and 2012**

**Variables**	**Crude OR**^**a**^	**Model 1**^**b **^**(n = 178038)**	**Model 2**^**b **^**(n = 178038)**	**Model 3**^**b **^**(n = 176145)**	**Model 4**^**b **^**(n = 148610)**
BMI^c^ <30.00	1	1	1	1	1
BMI ≥30.00	4.14 (3.81–4.50)	4.10 (3.74–4.42)	4.00 (3.68–4.35)	3.93 (3.62–4.28)	3.66 (3.31–4.01)
Age <35	1	1	1	1	1
Age ≥35	1.79 (1.15–1.95)	1.72 (1.58–1.87)	1.71 (1.57–1.86)	1.71 (1.57–1.86)	1.60 (1.59–1.93)
Nordic countries^d^	1		1	1	1
Non Nordic countries	2.24 (2.06–2.43)		2.18 (2.01–2.37)	2.10 (1.92–2.28)	2.10 (1.87–2.28)
Employed^e^	1			1	1
Unemployed^f^	1.74 (1.57–1.93)			1.27 (1.14–1.42)	1.20 (1.10–1.36)
University level^g^	1				1
<University level	1.62 (1.48–1.77)				1.34 (1.21–1.47)

^a^Crude odds ratio.

^b^Adjusted odds ratio and their 95% confidence intervals.

^c^Body mass index (BMI) kg/m^2^.

^d^Norway, Finland, Denmark, and Iceland.

^e^Employed includes employed, student, and parental leave.

^f^Unemployed includes \unemployed, sick leave, and other.

^g^Elementary school/high school.

### Pregnancy outcomes in relation to gestational diabetes mellitus

Maternal characteristics and pregnancy outcomes are presented in Table 3 in relation to the four screening regimes and the subcategories of GDM and non-GDM within each screening regime. The maternal BMI was significantly higher for GDM cases compared to non-GDM cases regardless of screening regime category. The highest mean BMI was found for GDM cases exposed to screening regime C (29.22 kg/m^2^) (Table 3). The prevalence of LGA was significantly higher for GDM cases in all screening regime categories. In relation to non-GDM cases in each screening regime category, the LGA prevalences were more than doubled in screening regimes A and B, more than three times higher in screening regime C, and five times higher in screening regime D (Table 3). The highest mean birth weight was demonstrated for GDM cases in screening regime C (3636 g) (Table 3). There was a significantly increased risk of CS delivery in the group of GDM cases compared with non-GDM cases (OR = 1.90, 95% CI 1.74-2.07). However, there was no statistically significant difference between elective CS or emergency CS regarding GDM (p = 0.169).

### Large for gestational age in relation to maternal characteristics

Large for gestational age fetus was strongly related to maternal obesity (BMI > 30), with an OR of 2.46 and 95% CI 2.33-2.60. When adjusting for maternal age, the OR for LGA moderately changed with an OR of 2.44 (95% CI 2.31-2.58). Furthermore, when adjusting for screening regime category, the OR was only slightly altered (OR 2.38, 95% CI 2.25-2.54). Non-Nordic origin demonstrated a protecting effect for LGA (OR 0.75; 95% CI 0.71-0.80).

## Discussion

Between 2011 and 2012 there was no national consensus regarding screening regimes of GDM in Sweden, and this situation is still prevailing. In this population-based cross-sectional study, we found that four screening and diagnostic regimes of GDM were applied in Sweden. The highest prevalence of OGTT (93.6%) was seen in the MHCA that used universal screening, and the lowest prevalence of OGTT was seen in the MHCA that used selective screening with an OGTT 2-hour cut-off value of 12.2 mmol/L as criteria for GDM. The highest prevalence of GDM (2.9%) was seen in the area where selective screening used an OGTT 2-hour cut-off value of 8.9 mmol/L. Maternal obesity, maternal age more than 35 years, non-Nordic origin, and lower level of education were all prominent risk factors for GDM. Furthermore, these background variables constituted risk factors for LGA except for women with a non-Nordic origin, resulting in a decreased risk of LGA. Most pregnant women in Sweden underwent selective screening of GDM using different OGTT 2-hour cut-off values for diagnosis. Few Swedish pregnant women (11.3%) were offered universal screening for GDM. In the MCHA with the lowest OGTT 2-hour cut-off value (8.9 mmol/L) as criteria for diagnosis of GDM, the prevalence of GDM was significantly higher (2.9%), compared to areas that used higher OGTT 2-hour cut-off values (10.0 and 12.2 mmol/L; 2.2% and 0.8%) as criteria for GDM diagnosis.

Sweden is considered to have a rather low prevalence of GDM (i.e., around 1.4%) [12]. Using the current screening methods and thresholds in Sweden, this study confirmed this level of GDM. Clearly, the “true level” of GDM within a specific setting depends on screening method (universal/general), the criterion for diagnosis of GDM [22], coverage of OGTT in the targeted population, the OGTT 2-hour cut-off value, and background characteristics of the pregnant population [23].

The International Association of Diabetes and Pregnancy Study Group (IADPSG) and WHO suggest the following diagnostic criteria and cut-off values for diagnosis of GDM: a fasting plasma glucose of 5.1 mmol/L (92 mg/dL) or a 1-hour plasma glucose of 10.0 mmol/L (180 mg/dL) or a 2-hour plasma glucose of 8.5 mmol/L (153 mg/dL) [18]. Only a minor part (4.7%) of Swedish pregnant women were exposed to a screening regime for diagnosing GDM at a 2-hour plasma glucose of 8.9 mmol/L, which is higher than the cut-off value of 8.5 mmol/L recommended by IADPSG and WHO [17,18]. Furthermore, more than one quarter of Swedish women underwent selective screening regime with a 2-hour plasma glucose value of 12.2 mmol/L or more, which commonly is graded as manifest diabetes mellitus. We find this selective screening regime unacceptable. The results in our study agree with the findings in the HAPO studies, where increasing 2-hour glucose values are associated with increasing risks for adverse pregnancy outcomes [2,6]. In fact, a major part of the world has adopted the recommended diagnostic criteria presented by IADPSG and WHO and our suggestion is that Sweden should follow this trend as well.

The resistance to accepting IADPSG and WHO recommendation of using the OGTT 2-hour cut-off values is related to expectations of increased work load for primary health providers due to the demand of follow-up by health services and the issue of unproven cost-effectiveness [24].

In our study, the highest prevalence of LGA (20.6%) was found in the MHCA where selective screening with a 2-hour cut-off value of 12.2 mmol/L for GDM diagnosis was applied. This high level of LGA differs from the prevalence of LGA in other MHCA where other diagnostic criteria for GDM were applied, such as a 2-hour cut-off value of 10.0 mml/L (universal screening; LGA prevalence of 10.5%) and selective screening with a 2-hour cut-off value of 8.9 mml/L (selective screening; LGA prevalence of 10.4%).

This high level of LGA (20.6%) might be explained by undiagnosed glucose intolerance with additional non-exposure to intervention by health care services. This situation is especially noteworthy since the mean maternal BMI (24.11 kg/m^2^) in this GDM screening regime category was significantly lower (p-value <0.001) compared to the mean maternal BMI in the other categories of GDM screening regimes (Table 3). The non-consensus regarding OGTT 2-hour cut-off values for diagnosis of GDM and the different regimes for screening is consistent with European protocols as a whole, demonstrating inconsistencies in GDM screening practices [23].

As demonstrated by the HAPO studies, there are continuous associations between maternal glucose levels and increasing birth weight and prevalence of caesarean section [6,14,25]. As previously mentioned, our study’s results agree with these results and demonstrate increased risks of LGA, instrumental vaginal delivery, and caesarean section in relation to increased levels of OGTT 2-hour glucose values. Increased maternal age as a risk factor for GDM has been reported previously [3,4], and our study confirms this association. Low socioeconomic status as a risk factor for GDM has also been reported previously [4], a finding also confirmed by our study. We suggest that older maternal age and low socioeconomic status should be considered as indicators for performance of OGTT when using a selective screening approach. Adverse pregnancy outcomes such as CS are well known as an adverse pregnancy outcome related to GDM, an association also confirmed by our study where the prevalence of CS increased with increased OGTT 2-hour glucose values. The OR was 1.92 times higher (95% CI 1.75-2.06) for CS compared to vaginal delivery for women with GDM compared to pregnant women without GDM. The same pattern could be seen regarding the risk of LGA. Women of non-Nordic origin presented a significant lower risk for LGA compared to women of Nordic origin (OR 0.75, 95% CI 0.71-0.80) and these findings have been reported previously [26].

The OR was 1.79 times higher (95% CI 1.15-1.95) for maternal age ≥35 years compared to maternal age <35 years. The importance of including maternal age of 35 years or more as an indicator for OGTT was supported by the PAPs values calculated for GDM for the two screening regimes “A” and “C” (see Results), showing that 14% vs. 17% could be attributed to maternal age ≥ 35 years.

Obesity is a global epidemic and has become a public health issue due to its association with complications during pregnancy, including significant adverse conditions such as preeclampsia, GDM, LGA, stillbirth, and caesarean section [27,28]. In our study, obese women had a substantial increased risk of GDM (OR 4.14; CI 95%: 3.81-4.50) compared to normal weight women.

### Methodological considerations

The internal validity of data included in the Maternal Health Care Register (MHCR) has been investigated. Unpublished results have shown an overall high coverage of variables and a satisfying quality of a major part of variables included in the register (personal communication). Because the coverage in the MHCR is considered high (81% in 2011 and 85% in 2012; personal communication), we believe the materials to be representative of the population of pregnant women in Sweden. Data on maternal height and maternal weight in the MHCR are mostly self-reported data (personal communication). Underreporting of the true maternal weight (i.e., reporting false lower weight) might have influenced the results, resulting in an underestimation of the prevalence of obesity in the Swedish pregnant population, and accordingly an underestimation of the associations related to BMI. However, we do not consider this situation as a major source of bias.

### Ethical considerations

All health national registers in Sweden, including MHCR, comply with the rules and procedures stated by The National Board of Health and Welfare in Sweden. Collection and management of patient data in health systems and health registers are regulated through the Swedish Patient Data Law. Participation in MHCR is voluntarily. Midwives in antenatal care inform pregnant women on the aims and participation of MHCR using the following strategies: a) advertisements in the waiting room at the antenatal clinic, b) written information provided if requested by the pregnant woman, and c) at registration in antenatal care the pregnant woman receives information orally from the midwife on how data are documented in the medical files and in MHCR. In a majority of the MHCA, a pregnant woman completes a form on health issues before registration in antenatal care. Furthermore, the eligible participants receive information that all results will be presented on an aggregated level, so no individual subject can be identified.

## Conclusions

There was no consensus regarding screening regimes for GDM in Sweden. Increasing levels of OGTT 2-hour glucose values were strongly associated with adverse pregnancy outcomes. Applying different screening regimes and definitions of GDM in Sweden results in different procedures in clinical management of pregnant women which contributes to unequal health care. Using 2-hour capillary glucose does not follow international recommendations. We therefore suggest that Sweden adopts the recent recommendations outlined by IADPSG concerning the performance of OGTT and the diagnostic criteria for GDM. The results in our study further support that maternal age ≥35 years of age and educational level should be regarded as risk indicators for performance of OGTT during pregnancy.

## Competing interests

The authors declare that they have no competing interests.

## Authors’ contributions

All authors have sufficiently contributed to this study. ML, IM, MP, and MLT designed the study. ML collected the data, conducted the first analyses, and drafted the manuscript in close collaboration with IM. ML and IM performed the statistical analyses with assistance from MLT. MP and MLT contributed to the manuscript during the work process. All authors have read and approved the final manuscript.

## Pre-publication history

The pre-publication history for this paper can be accessed here:

http://www.biomedcentral.com/1471-2393/14/185/prepub
